# Supplementary figures and images for: The role of hypermutation and collateral sensitivity in antimicrobial resistance diversity of Pseudomonas aeruginosa populations in cystic fibrosis lung infection
Source: mBio. 2024 Jan 3;15(2):e03109-23. doi: 10.1128/mbio.03109-23 (PMC10865868; doi:10.1128/mbio.03109-23)

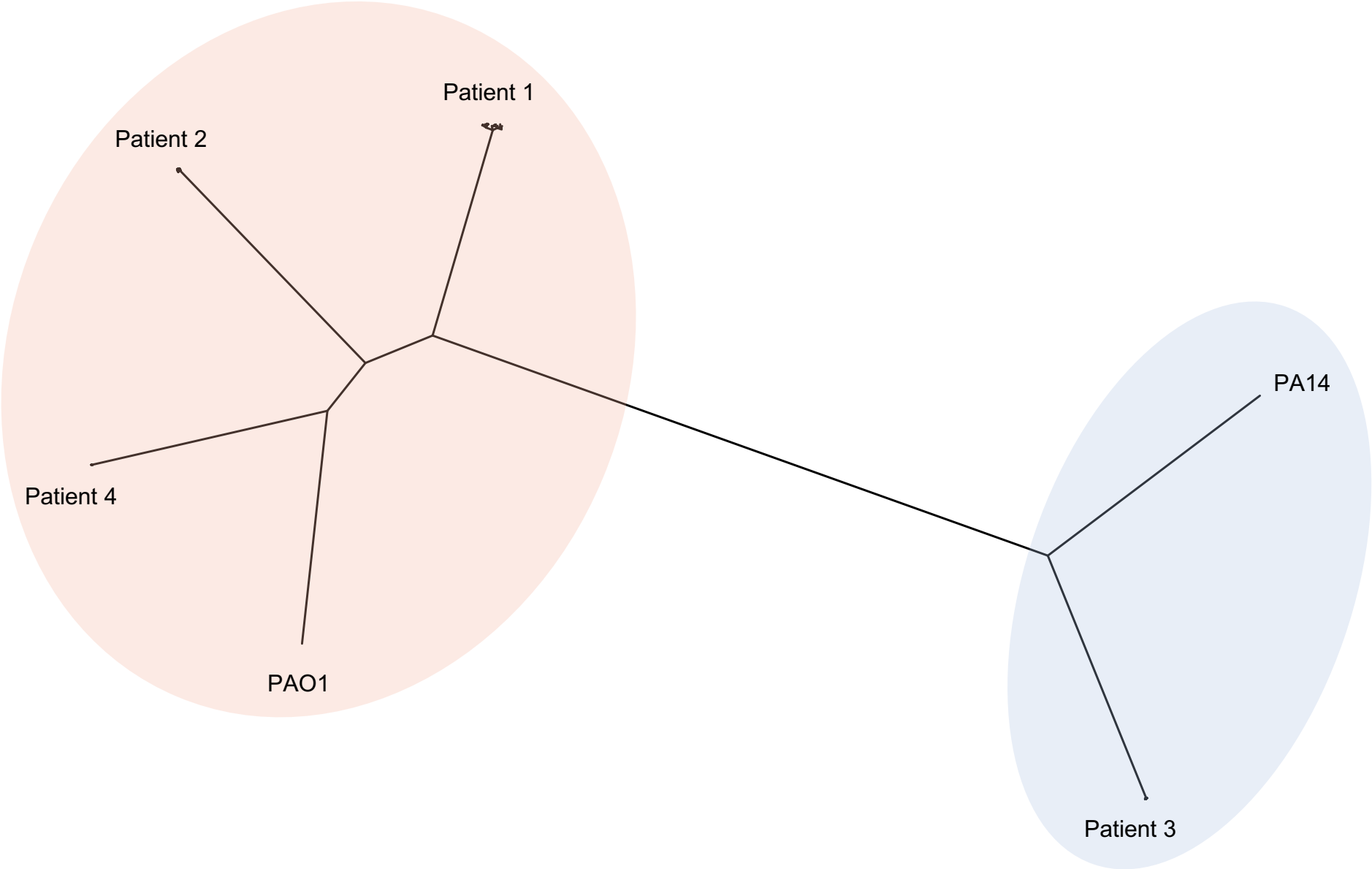

Supplement: Fig. S1 — Phylogeny of patients 1-4 with PAO1 and PA14. [file mbio.03109-23-s0001.pdf]

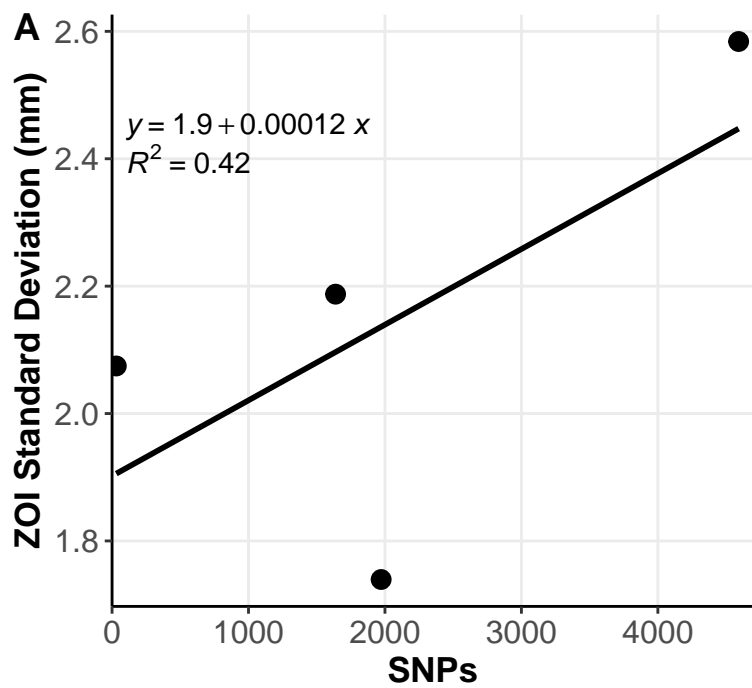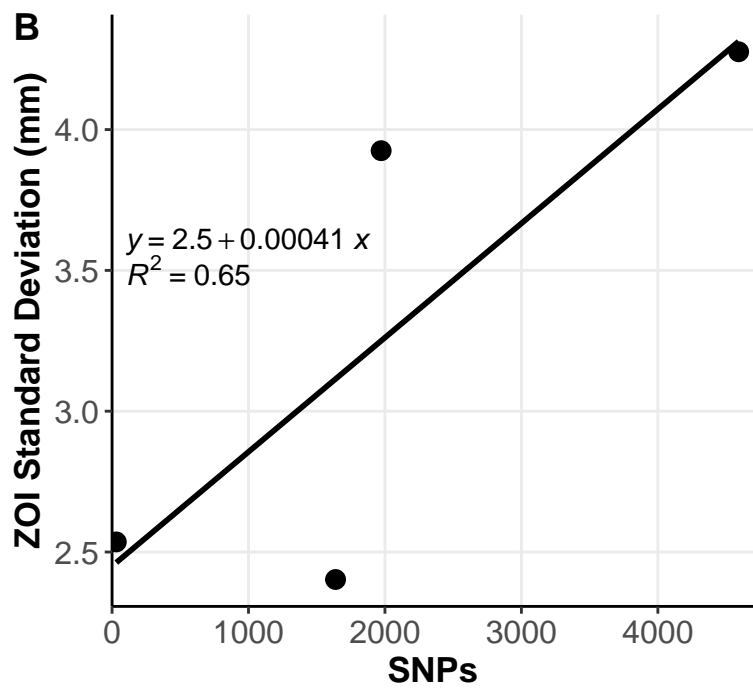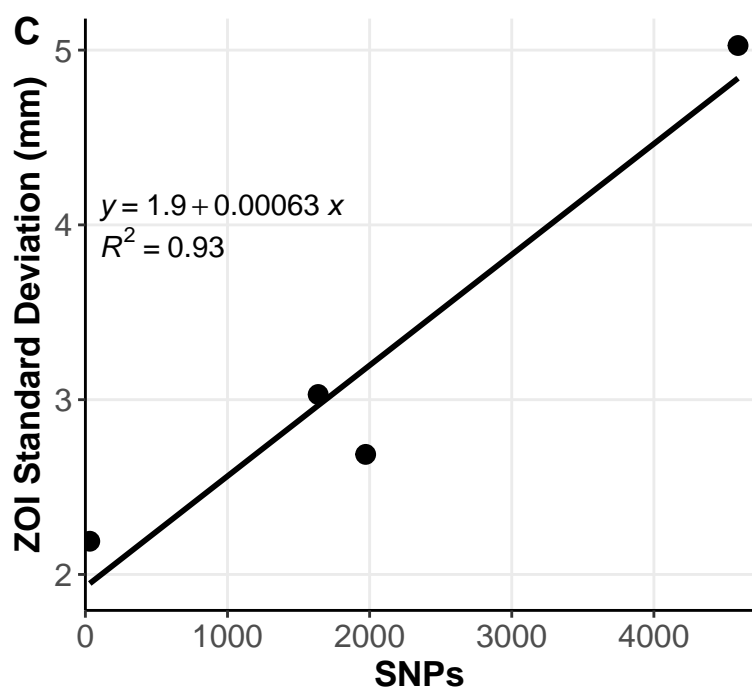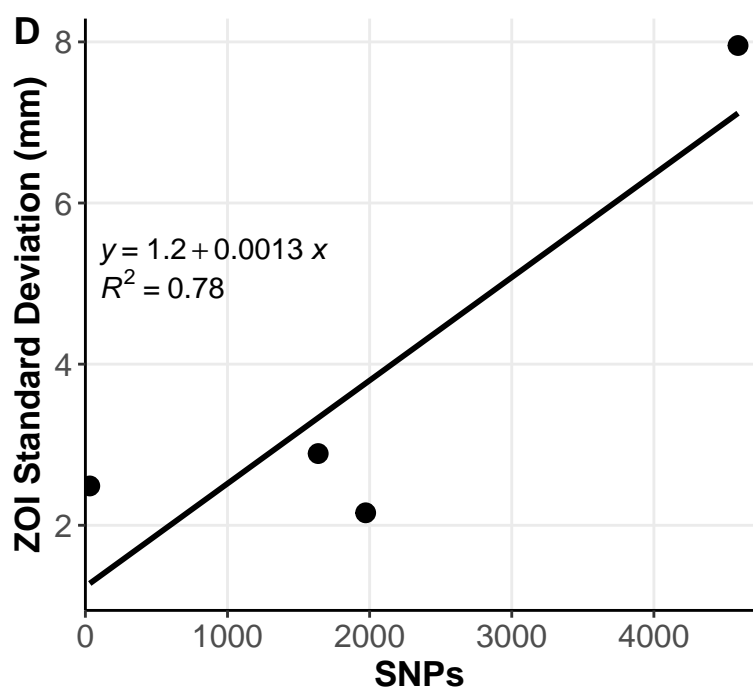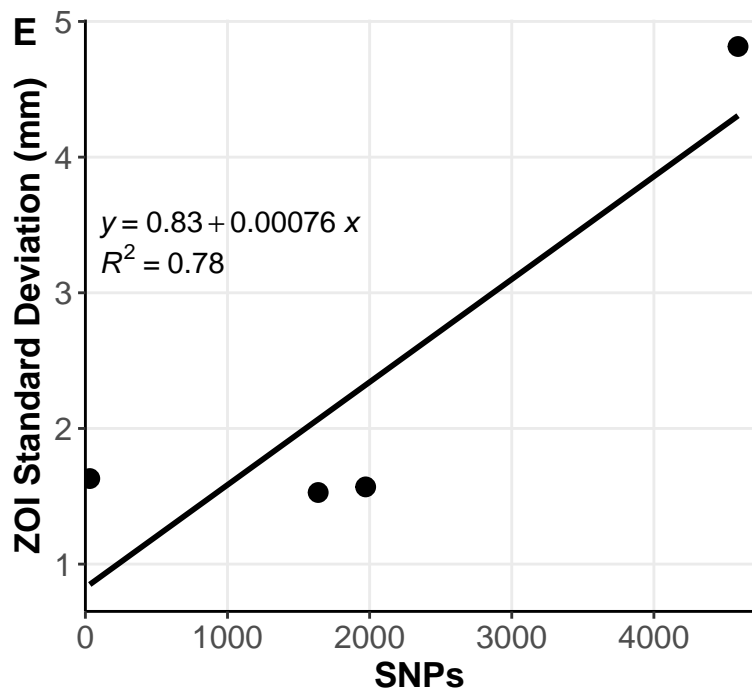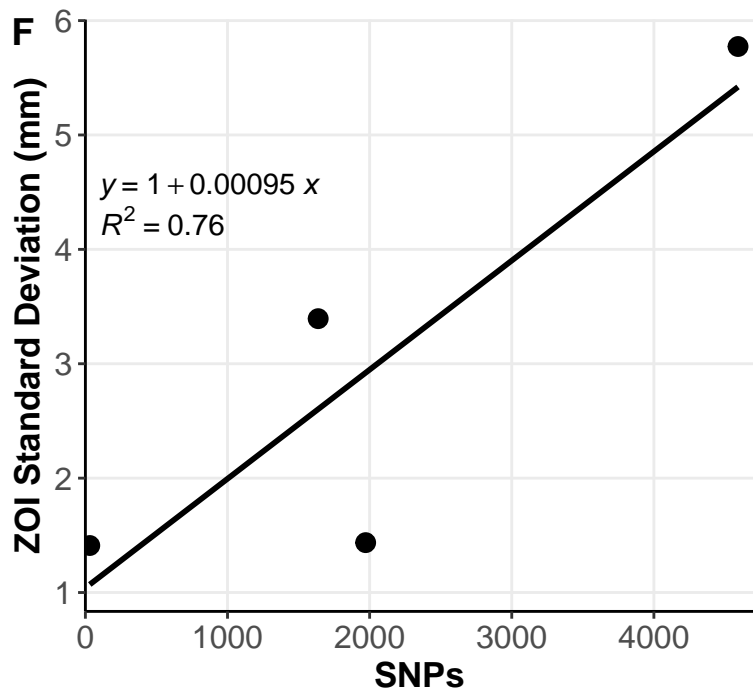

Supplement: Fig. S2 — Linear regression analysis of total SNP count as an indicator of AMR diversity. [file mbio.03109-23-s0002.pdf]

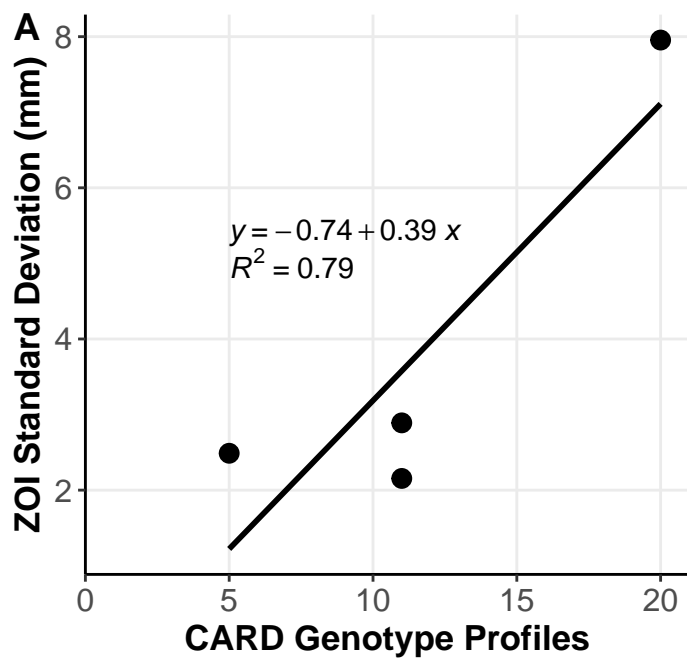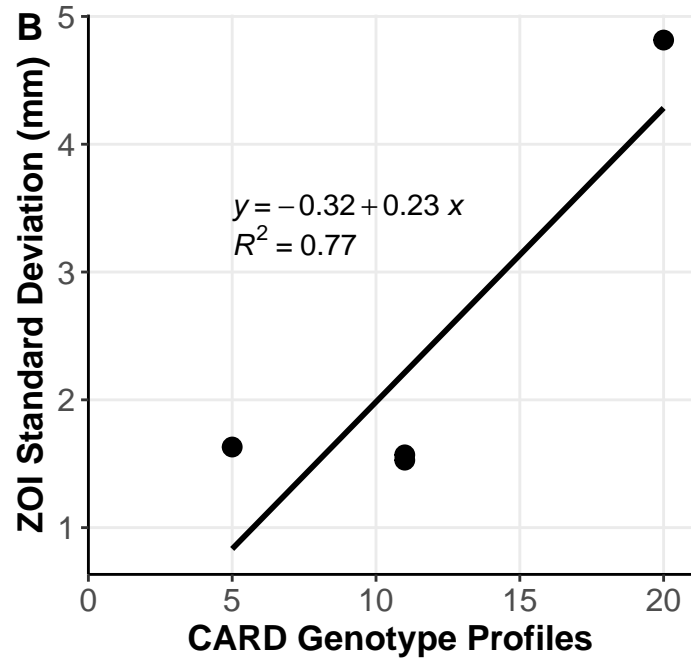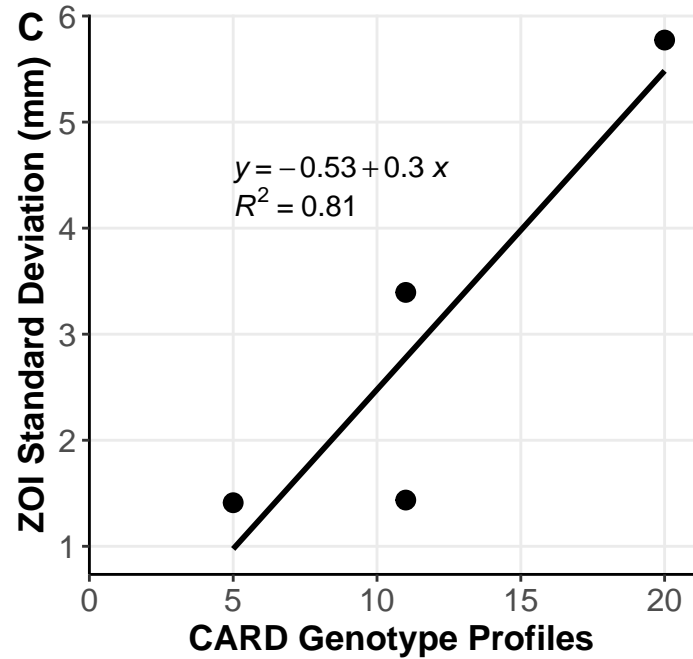

Supplement: Fig. S3 — Linear regression analysis of number of distinct CARD profiles within a population as a predictor of population standard deviation. [file mbio.03109-23-s0003.pdf]

A

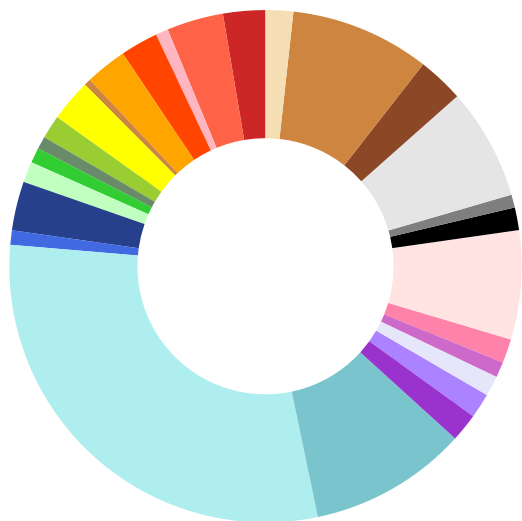

B

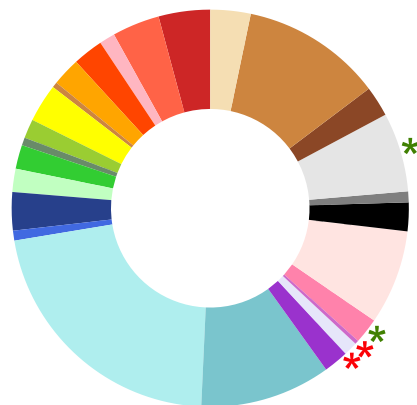

C

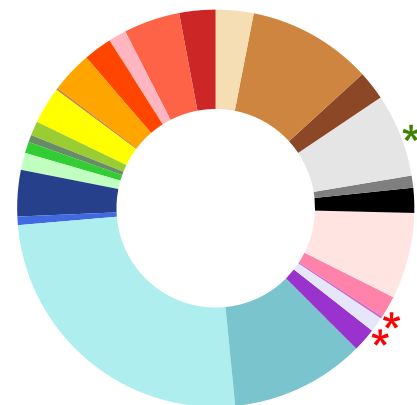

D

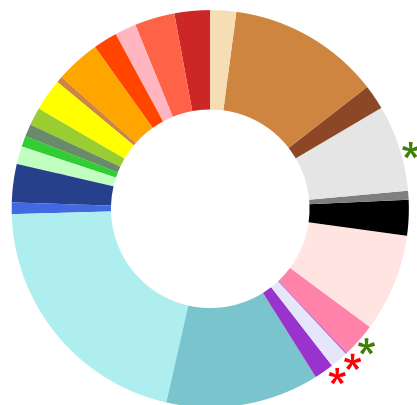

E

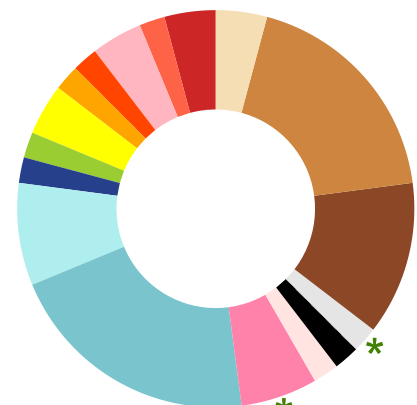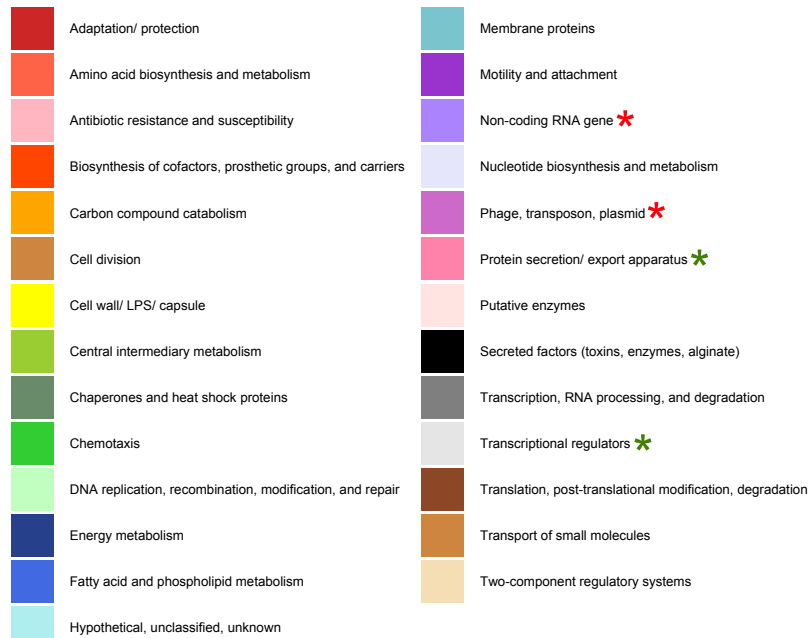

Supplement: Fig. S4 — Enrichment analysis of the frequency of functional categories in which non-synonymous SNPs and microindels are found. [file mbio.03109-23-s0004.pdf]

**Patient 1**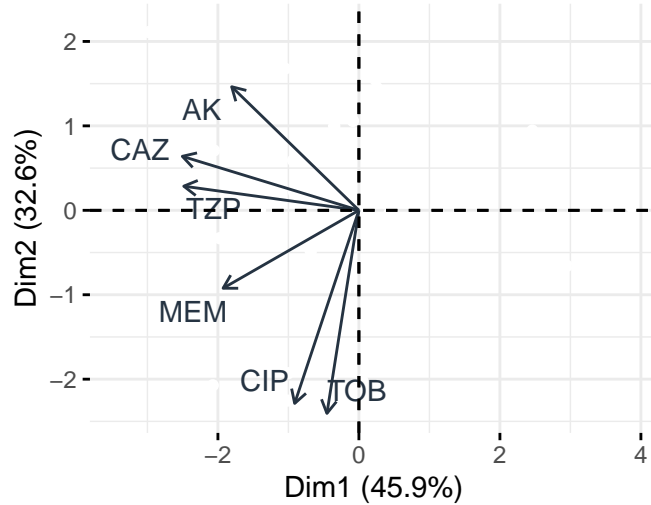**Patient 2**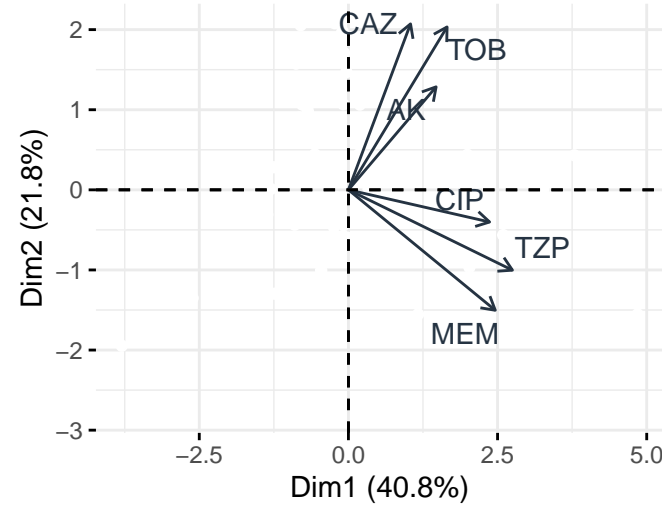**Patient 3**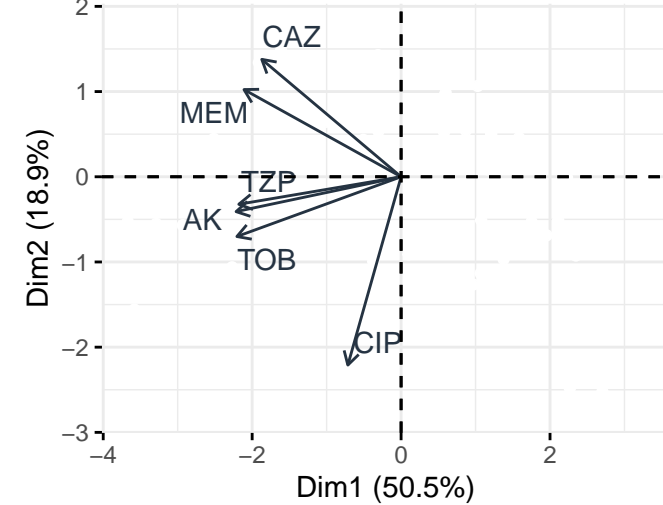**Patient 4**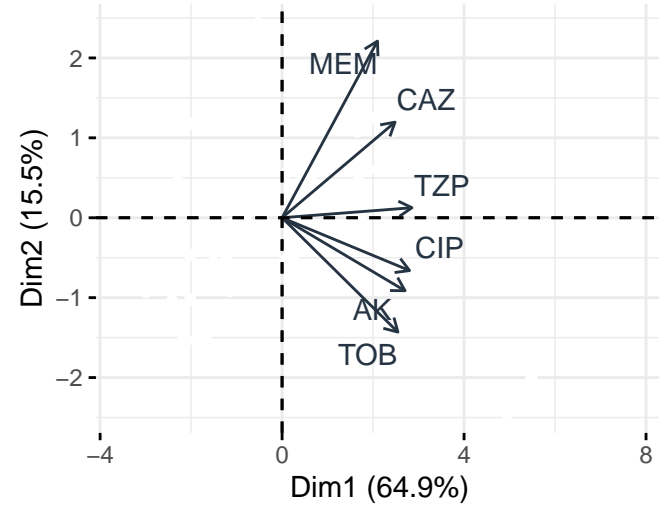

Supplement: Fig. S5 — Principal components analysis vectors display no evidence of collateral sensitivity across any of the six antimicrobials tested for any patient. [file mbio.03109-23-s0005.pdf]

Patient 1 2 3 4

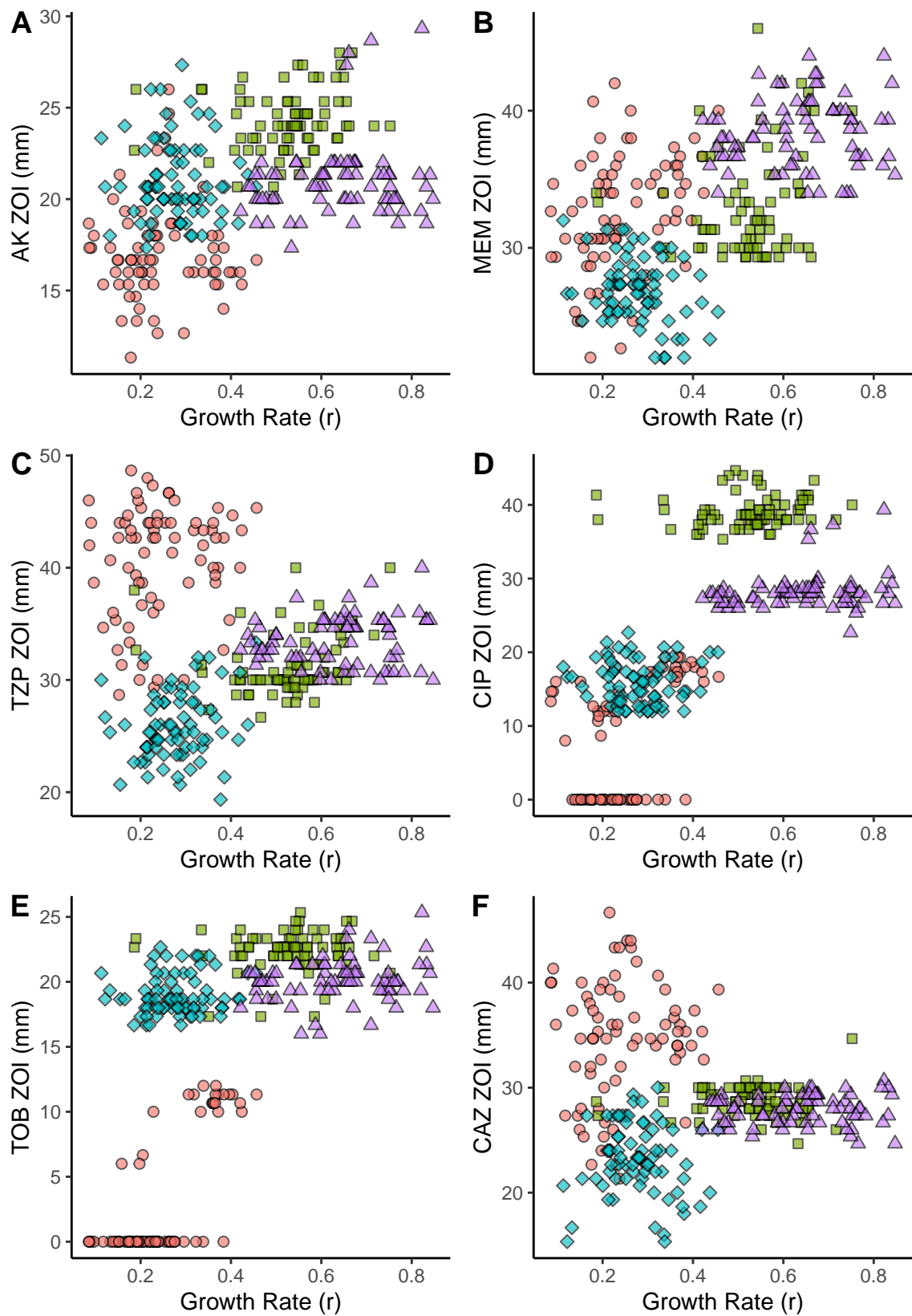

Supplement: Fig. S6 — Scatterplots of zone of inhibition versus growth rate in SCFM for all six tested antibiotics. [file mbio.03109-23-s0006.pdf]
