# Supplementary material for: The role of hypermutation and collateral sensitivity in antimicrobial resistance diversity of Pseudomonas aeruginosa populations in cystic fibrosis lung infection
Source: mBio. 2024 Jan 3;15(2):e03109-23. doi: 10.1128/mbio.03109-23 (PMC10865868; doi:10.1128/mbio.03109-23)
Supplement: Supplemental legends — Legends for supplemental figures and tables. [file mbio.03109-23-s0007.docx]

**Supplemental figure legends**

**Supplemental Figure 1.** Phylogeny of Patients 1-4 with PAO1 and PA14. Patients 1, 2, and 4 cluster with PAO1, while Patient 3 clusters with PA14.

**Supplemental Figure 2.** Linear regression analysis demonstrates that total SNP count in a population was a strong indicator of AMR diversity for amikacin (R^2^ = .90, F(1, 2) = 18.94, *p* = .049), meropenem (R^2^ = .93, F(1, 2) = 25.3, *p* = .037), and piperacilin-tazobactam (R^2^ = .95, F(1, 2) = 39.86, *p* = .024), but a poor indicator of AMR diversity for ciprofloxacin (R^2^ = .12, F(1,2) = .27, *p* = .65) and ceftazidime (R^2^ = .71, F(1,2) = 4.78, *p* = .16), and was inversely related to AMR diversity for tobramycin (R^2^ = .97, F(1,2) = 66.61, *p* = .015)

**Supplemental Figure 3.** Linear regression analysis shows that the number of distinct CARD profiles within a population is an improved predictor of population standard deviation for ciprofloxacin (R^2^ = .79, F(1,2) = 7.35, *p* = .11), tobramycin (R^2^ = .77, F(1,2) = 6.73, *p* = .12), and ceftazidime (R^2^ = .81, F(1,2) = 8.44, *p* = .10) over total population SNP count.

**Supplemental Figure 4.** Enrichment analysis of the frequency of functional categories in which non-synonymous SNPs and microindels are found in each of the four populations relative to the proportions of these functional categories in the PAO1 genome shows that protein secretion/ export apparatuses and transcriptional regulators are enriched for such variants, while phage/ transposon/ plasmid and non-coding RNA are less likely to be impacted by such variants. Donut plot of the relative frequencies of genes categorized within each of the 27 different PseudoCAP functional categories in the PAO1 genome (A). Donut plots of the relative frequencies of non-synonymous SNPs and indels located in each of the 27 different PseudoCAP functional categories in Patient 1 (B), 2 (C), 3 (D), and 4 (E). Protein secretion/ export apparatuses and transcriptional regulators are denoted with green asterisks on donut plots where applicable, while phage/ transposon/ plasmid and non-coding RNA are denoted with red asterisks.

**Supplemental Figure 5.** Principal components analysis vectors display no evidence of collateral sensitivity across any of the six antimicrobials tested for any patient, and further demonstrate that cross-resistance and cross-sensitivity patterns differ across patients.

**Supplemental Figure 6.** Scatterplots of zone of inhibition (ZOI) versus growth rate (r) in SCFM for all six tested antibiotics: amikacin (AK), meropenem (MEM), piperacillin-tazobactam (TZP), ciprofloxacin (CIP), tobramycin (TOB), and ceftazidime (CAZ). Results of linear mixed model (Table S18), with growth rate in SCFM as a fixed effect and patient as a random effect, demonstrate that there is no significant effect of growth rate on resistance, and therefore, no evidence for trade-offs between growth rate and resistance in these four populations.

**Supplemental Table 1.** Antimicrobial susceptibility testing measurements for Patient 1 as measured by zone of inhibition (ZOI) in a standard disc diffusion assay for amikacin (AK), meropenem (MEM), piperacilin-tazobactam (TZP), ciprofloxacin (CIP), tobramycin (TOB), and ceftazidime (CAZ). Data in the left columns represent raw measurements of zone of inhibition radii (mm units). Data in the right columns represent calculated zone of inhibition values as diameters (mm units).

**Supplemental Table 2.** Antimicrobial susceptibility testing measurements for Patient 2 as measured by zone of inhibition (ZOI) in a standard disc diffusion assay for amikacin (AK), meropenem (MEM), piperacilin-tazobactam (TZP), ciprofloxacin (CIP), tobramycin (TOB), and ceftazidime (CAZ). Data in the left columns represent raw measurements of zone of inhibition radii (mm units). Data in the right columns represent calculated zone of inhibition values as diameters (mm units).

**Supplemental Table 3.** Antimicrobial susceptibility testing measurements for Patient 3 as measured by zone of inhibition (ZOI) in a standard disc diffusion assay for amikacin (AK), meropenem (MEM), piperacilin-tazobactam (TZP), ciprofloxacin (CIP), tobramycin (TOB), and ceftazidime (CAZ). Data in the left columns represent raw measurements of zone of inhibition radii (mm units). Data in the right columns represent calculated zone of inhibition values as diameters (mm units).

**Supplemental Table 4.** Antimicrobial susceptibility testing measurements for Patient 4 as measured by zone of inhibition (ZOI) in a standard disc diffusion assay for amikacin (AK), meropenem (MEM), piperacilin-tazobactam (TZP), ciprofloxacin (CIP), tobramycin (TOB), and ceftazidime (CAZ). Data in the left columns represent raw measurements of zone of inhibition radii (mm units). Data in the right columns represent calculated zone of inhibition values as diameters (mm units).

**Supplemental Table 5.** Genome size, average sequencing coverage, and number of contigs of each assembly.

**Supplemental Table 6.** Supporting statistical values of the linear regression analysis of distinct CARD resistance genotype profiles within a population as a proxy for genomic diversity as measured by total SNPs in each population.

**Supplemental Table 7.** Genes that were impacted by non-synonymous mutations in at least one isolate in all four populations.

**Supplemental Table 8.** Full details of the chi-squared goodness of fit and Monte Carlo simulation exact multinomial tests, with all associated chi-squared and p-values.

**Supplemental Table 9.** Genes that were impacted by non-synonymous mutations in at least one isolate in three out of four populations.

**Supplemental Table 10.** All annotated genetic variants discovered in Patient 1.

**Supplemental Table 11.** All annotated genetic variants discovered in Patient 2.

**Supplemental Table 12.** All annotated genetic variants discovered in Patient 3.

**Supplemental Table 13.** All annotated genetic variants discovered in Patient 4.

**Supplemental Table 14.** Raw OD_600_ reads for growth in SCFM used to create growth curves and analyze growth rate (r) for Patient 1. Time is given in hours, and all isolates were tested in biological triplicates.

**Supplemental Table 15.** Raw OD_600_ reads for growth in SCFM used to create growth curves and analyze growth rate (r) for Patient 2. Time is given in hours, and all isolates were tested in biological triplicates.

**Supplemental Table 16.** Raw OD_600_ reads for growth in SCFM used to create growth curves and analyze growth rate (r) for Patient 3. Time is given in hours, and all isolates were tested in biological triplicates.

**Supplemental Table 17.** Raw OD_600_ reads for growth in SCFM used to create growth curves and analyze growth rate (r) for Patient 4. Time is given in hours, and all isolates were tested in biological triplicates.

**Supplemental Table 18.** Supporting brms R code and statistical values for the linear mixed model run to assess the relationship between growth rate (r) and antimicrobial resistance. Results of the model, with growth rate in SCFM as a fixed effect and patient as a random effect, show that the 95% confidence interval of the fixed effect of growth rate spans 0 for all six antimicrobials. Therefore, the null hypothesis that the fixed effect of growth on antimicrobial susceptibility is 0 cannot be rejected, providing no evidence for trade-offs or any significant relationship between resistance and growth rate across all four populations.
